# Time course of traumatic neuroma development

**DOI:** 10.1371/journal.pone.0200548

**Published:** 2018-07-16

**Authors:** Karla M. C. Oliveira, Lukas Pindur, Zhihua Han, Mit B. Bhavsar, John H. Barker, Liudmila Leppik

**Affiliations:** Frankfurt Initiative for Regenerative Medicine, JW Goethe-University, Frankfurt/Main, Germany; University Hospital Wurzburg, GERMANY

## Abstract

This study was designed to characterize morphologic stages during neuroma development post amputation with an eye toward developing better treatment strategies that intervene before neuromas are fully formed. Right forelimbs of 30 Sprague Dawley rats were amputated and limb stumps were collected at 3, 7, 28, 60 and 90 Days Post Amputation (DPA). Morphology of newly formed nerves and neuromas were assessed via general histology and neurofilament protein antibody staining. Analysis revealed six morphological characteristics during nerve and neuroma development; 1) normal nerve, 2) degenerating axons, 3) axonal sprouts, 4) unorganized bundles of axons, 5) unorganized axon growth into muscles, and 6) unorganized axon growth into fibrotic tissue (neuroma). At early stages (3 & 7 DPA) after amputation, normal nerves could be identified throughout the limb stump and small areas of axonal sprouts were present near the site of injury. Signs of degenerating axons were evident from 7 to 90 DPA. From day 28 on, variability of nerve characteristics with signs of unorganized axon growth into muscle and fibrotic tissue and neuroma formation became visible in multiple areas of stump tissue. These pathological features became more evident on days 60 and 90. At 90 DPA frank neuroma formation was present in all stump tissue. By following nerve regrowth and neuroma formation after amputation we were able to identify 6 separate histological stages of nerve regrowth and neuroma development. Axonal regrowth was observed as early as 3 DPA and signs of unorganized axonal growth and neuroma formation were evident by 28 DPA. Based on these observations we speculate that neuroma treatment and or prevention strategies might be more successful if targeted at the initial stages of development and not after 28 DPA.

## Introduction

Limb amputation is a devastating condition caused by trauma and diseases, resulting in both functional and psychological disability in affected patients [1,2]. About 61% of amputee patients report residual limb pain, of which 48.7% are estimated to be caused by a sensitized neuroma [3]. Neuroma induced pain can also occur as a result of lacerations, crush injuries, and in peripheral nerves after microtrauma from stretching or compression of local tissues [4].

Neuromas are typical, benign neural tumors that occur after nerve injury, however the signaling mechanisms and development pathways involved in the formation of these lesions are poorly understood [5]. Researchers have demonstrated that inhibiting Nerve Growth Factor (NGF) after nerve injury reduces neuroma formation and neuropathic pain in rat models [6]. Also, local deactivation of Brain Derived Nerve Factor (BDNF) has been shown to significantly reduce neuropathic pain and affect regeneration of sensory fibers, while high concentrations of BDNF increases neuroma and neuropathic pain development [7].

Several studies have shown that growth factors and signaling play an important role during nerve regeneration [8–10]. Following peripheral nerve injury, distal axons experience Wallerian degeneration, then via endocrine and paracrine signaling, proximal axons begin to grow to reconstruct nerve continuity (reviewed in [11,12]). However, the regenerative capacity of axons and growth support of Schwann cells (SCs) decline with time and distance from an injury (reviewed in [13]). When the distance between two severed injured nerve segments is long or if no distal end (amputations) is present, axon regrowth occurs in an unorganized pattern (reviewed in [11,14]). In these cases simultaneous proliferation of wound-repairing cells and signaling molecules can lead to collagen remodeling and scar formation that form poorly vascularized dense fibrous structures known as neuromas [15].

Histologically, traumatic neuroma can be characterized as non-encapsulated, non-neoplastic conglomerates of cells and axons embedded in a dense fibrotic matrix [16,17]. Some studies and case reports have described traumatic neuroma presenting tangled morphology, composed of connective tissue, Schwann cells, and regenerating axons [17–21]. Inflammatory signaling factors [20,21] and myofibroblasts [22] have also been identified in painful neuromas. While microscopic features of fully formed neuroma have been well documented, little information exists about the cellular structure of neuroma in their early stages of development, from the time of nerve injury through to complete tumor formation. This information could be used to develop new and better treatments that target the early stages of neuroma development and thus prevent the associated pain.

Many treatment options exist for painful neuroma. Pharmacological treatments include N-methyl-D-aspartate (NMDA) receptor antagonists, opioids, anticonvulsants, antidepressants, local anesthetics, and calcitonin, which act by inhibiting pain-signaling pathways. When using these treatments, chronic and adverse effects, and route of administration must be taken into consideration (reviewed in [23]). Invasive treatments are also used, like surgical neuroma resection although in most cases this approach is not successful [24]. Other methods used to treat and/or prevent neuroma formation at the time of injury, include targeted nerve implantation [25], collagen nerve wrapping [22,26], and transpositions into muscles [27] and veins [28]. Different combinations of these techniques are used and have been shown to be more effective for treating peripheral nerve injuries, minimizing neuroma formation, reducing pain, and diminishing phantom symptoms, compared to traction neurectomy alone [29]. While some of these treatments or combination of treatments show varying degrees of success, overall, existing treatments for painful neuromas remain unsatisfactory and many patients continue to suffer with this condition (reviewed in [30]). It is with this in mind that we embarked upon this study to try to better understand how a neuroma forms in the first place.

This study was designed to characterize the early histological stages of nerve regrowth and neuroma formation following limb amputation. In a rat limb amputation model we used histological and immunohistochemical (IHC) analysis to characterize neuroma development at different time points from the time of injury to full neuroma formation. We hope that this information will help to identify stages of neuroma development to target new and improved treatments that help alleviate pain and suffering in millions of patients worldwide.

## Materials and methods

All animal experiments were performed in accordance with guidelines established by our animal care and oversight committee at the Johann Wolfgang Goethe-University in Frankfurt/Main, Germany and were approved by the Veterinary Department of the Regional Council in Darmstadt, Germany (Regierungspräsidium Darmstadt, Veterinärdezernat, Wilhelminenstraße 1–3) (FU/1114) in accordance with German law.

Nerve regrowth and neuroma formation were assessed in the stumps of amputated limbs of 30 male Sprague Dawley rats (Charles River Labs Int., Germany) (age = 5 weeks; weight = 100–150g) at 3, 7, 28, 60 and 90 days after surgery (n = 6) using histological and immunohistochemistry analysis.

### Limb amputation surgery

All animals received prophylactic antibiotics (0,2 ml procaine penicillin containing 60.000U) prior to surgery. While under intraperitoneal general anesthesia (10 mg Ketamine/100mgBW; 1 mg Xylazine/100mgBW) the rats`right limbs were shaved and cleaned with antiseptic fluid and a circumferential skin incision was made over the elbow. After dissection of the subcutaneous tissue the brachial artery and accompanying veins were identified and ligated. The ulnar, median, and radial nerves were dissected free and divided and the muscles of the forelimb were cut 1cm proximal to the elbow joint. Only the right limb humerus was cut with a gigli saw wire. Hemostasis was achieved through short light compression. After amputation, the skin was closed over the limb stump with continuous intradermal suture (4–0 Prolene, Ethicon, Germany) and spray dressing (OPSITE Spray, Smith&Nephew, Canada) was applied on the wound. Animals were monitored postoperatively until they recovered from anesthesia, and then daily for complications and/or signs of pain and discomfort.

Animals were housed in separate cages in a light (12 hr light– 12 hr dark), temperature (20–24° C) and airflow controlled room and were given free access to food and water. At 3, 7, 28, 60, and 90 days groups of animals were euthanized (CO_2_ inhalation), weighed and limb stumps were collected and examined macro- and microscopically for signs of infection or tumors. After animals were euthanized, the contralateral (left) limbs of four animals were also collected. Since these nerves should present histologically normal appearance tissues from the left limbs served as baseline controls. All specimens were fixed in Zinc-Formal-Fixx (Thermo scientific, USA) for 24 hours and stored for subsequent histomorphometric and immunohistological analysis.

### Assessment of neuroma formation

#### Histology

To perform histological analysis amputated limb stump tissues were fixed, decalcified in 10% EDTA/TRIS-HCl (pH 7.4) for 14 days, then embedded in paraffin blocks. Representative longitudinal sections (5μm thick) were cut with a Microtome (Leica RM2235) and mounted on glass microscope slides.

***General morphology*:** Stump tissues from each time point were stained (Alcian Blue/Orange G-Hematoxilin-Eosin (AB&OG)) for morphological analysis [31], as described previously [32]. Evaluation of nerve structures was made using light microscopy (Large image scanning, Ti-E, Nikon GmbH, Germany) and image analysis software (NIS-Elements 4.4, Nikon GmbH, Germany).

***Immunohistochemistry (IHC)*:** To assess nerve regrowth in limb stumps by IHC, heat antigen retrieval was performed in Citrate Buffer (DAKO) for 10 min. After blocking with 7% goat serum solution (Dako, Germany), specimens were incubated with monoclonal mouse anti-human Neurofilament protein antibody (NF)(Clone 2F11, culture supernatant, 1:100; DAKO, Germany), which serves as a marker for axons. For signal detection, an EnVision + System-HRP (AEC) kit (Dako, Germany) was applied following the manufacturers`instructions. Finally, a counterstain with hematoxylin was performed and stained samples were analyzed using light microscopy (Large image scanning, Ti-E, Nikon GmbH, Germany). Morphological observations and quantitative evaluations were performed using image analysis software (NIS-Elements 4.4, Nikon GmbH, Germany). To assure that positive stain was not caused by non-specific interactions of immunoglobulin molecules, isotype control was carried out in representative samples.

**Fig 1:**
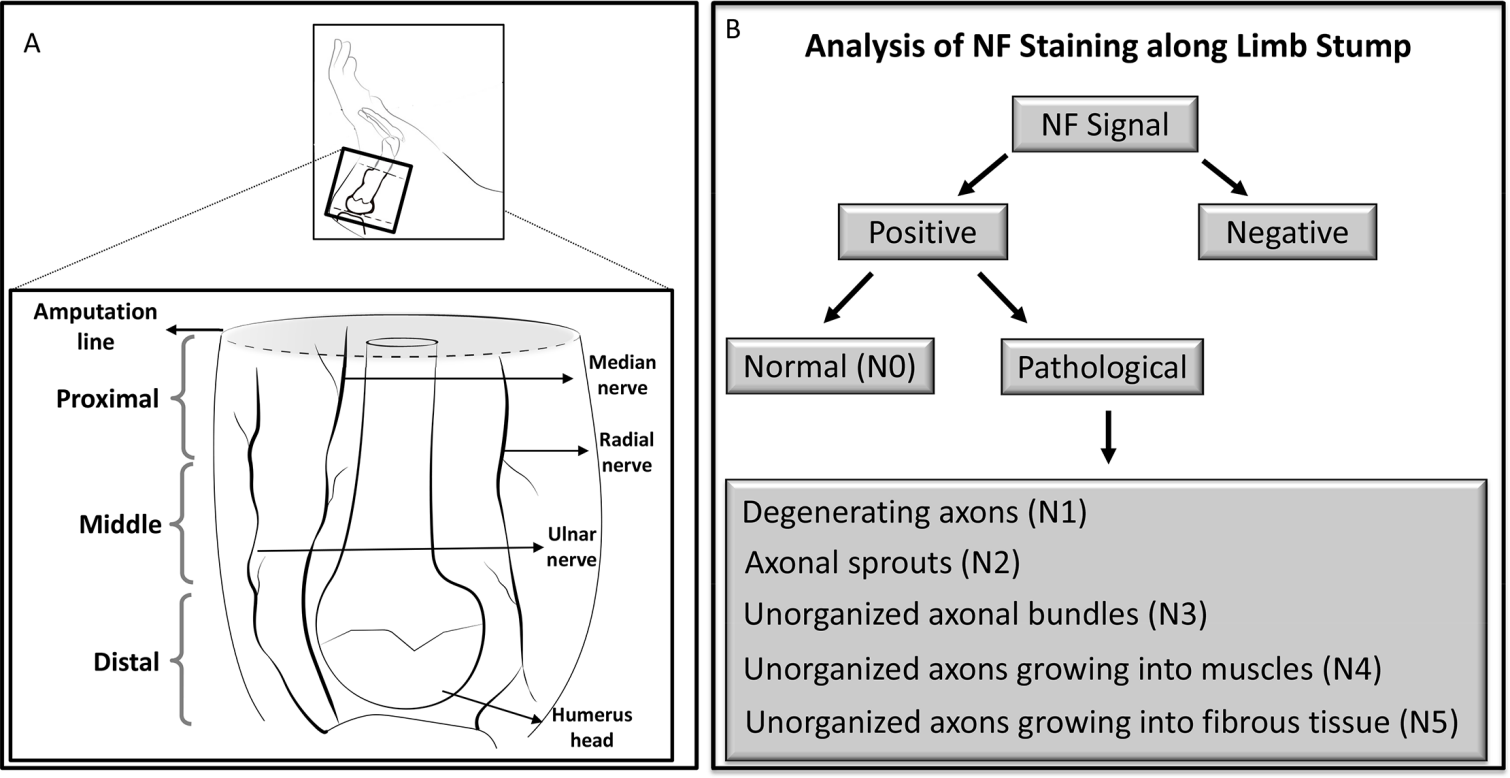
Schematic view of amputation site and parameters analyzed. (A) Representative amputation area of the rat limb and stump samples collected for analysis. (B) Schematic description of parameters analyzed.

***Evaluation parameters*:** To avoid variability in data interpretation one trained examiner evaluated all sections. Six limb stumps for each time point were analyzed and after careful examination, representative sections from each stump were taken for detailed evaluation. The stump sections obtained were evaluated from the humerus head to the most distal area of the amputated stump; thus assuring observations throughout the entire stump section (Fig 1A).

At each time point a qualitative scoring scheme was used, in which the presence and location of nerves was recorded as either positive or negative by NF labeling. Axonal density was measured to differentiate normal and degenerating axons. First the total area (region of interest–ROI) to be analyzed was selected then using a threshold tool to highlight NF positive stain in the ROI a value for the stained area was obtained. Axonal density was calculated as the area of stained axons in the fascicles divided by the total area in the ROI. In each sample, 2–3 fascicles and/or bundles detected in the stump section was measured at 20x magnification. Then mean values of axonal density of normal and degenerating axons, in each animal, were obtained for all time points.

The contrast between normal and pathological axonal distribution was detected by careful examination of the relation between nerve fascicles and axons and the surrounding tissues at different time points. The different axonal morphological structures were classified into 6 different categories as: Normal nerves–N0; Degenerating axons–N1; Axonal sprouts–N2; Unorganized bundles into conjunctive tissue–N3; Unorganized axons into muscles–N4; and Unorganized axons into fibrotic tissue—N5. Then their presence in the limb stump was cataloged in all samples for each time point (Fig 1B).

### Statistical analysis

Differences of axonal density in normal and degenerating axons at the different time points were analyzed by Wilcoxon Signed-Rank Test using Graphpad Prism software (p≤0.05). Bootstrap of the data from percentage of animals affected by each feature at each time point was performed with 1000 repetitions using R-Studio platform for R-software and 95% confidence intervals (95% CI) were later calculated.

## Results

### Histological characterization of nerves in limb stump tissue

**Fig 2:**
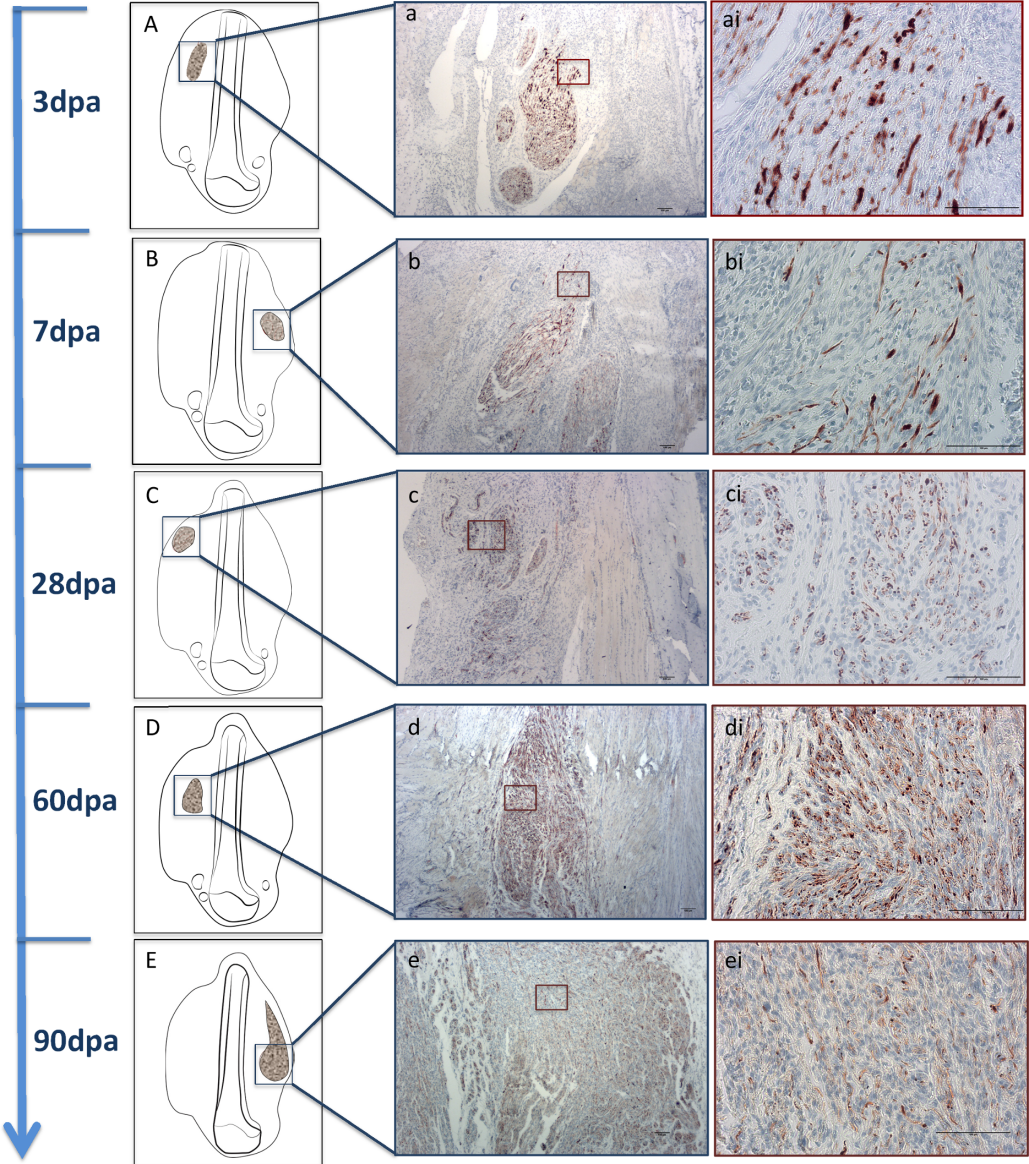
Specific NF staining of nerve regrowth and neuroma development observed in the tissue sections from day 3 until 90. (A, B, C, D and E) Schematic figures representing an overview of structures location and representative images of axonal fascicles show larger and closer view (a, b, c, d and e with 4x magnification; ai, bi, ci, di and ei with 20x magnification, scale bar = 100μm) of structures throughout time.

All stumps were sectioned longitudinally, along the long axis of the limb, to maximize the presence of nerves. Peripheral nerves within muscle and connective tissue were observed in all stumps, at all time points. Limb stumps were examined and the 6 morphological categories were assigned by overlapping AB&OG and NF-stained sections. AB&OG stained axon fascicles appeared light pink in color, surrounded by a slightly light brown colored membrane (perineurium) (in S1 Fig). Individual axons could only be identified at 20x magnification appearing as darker pink colored dots or sprouts. Detailed focal labeling of axons was clearly visible and readily distinguishable using immunohistochemistry with specific antibodies targeting neurofilament protein (NF)(Fig 2).

**Fig 3:**
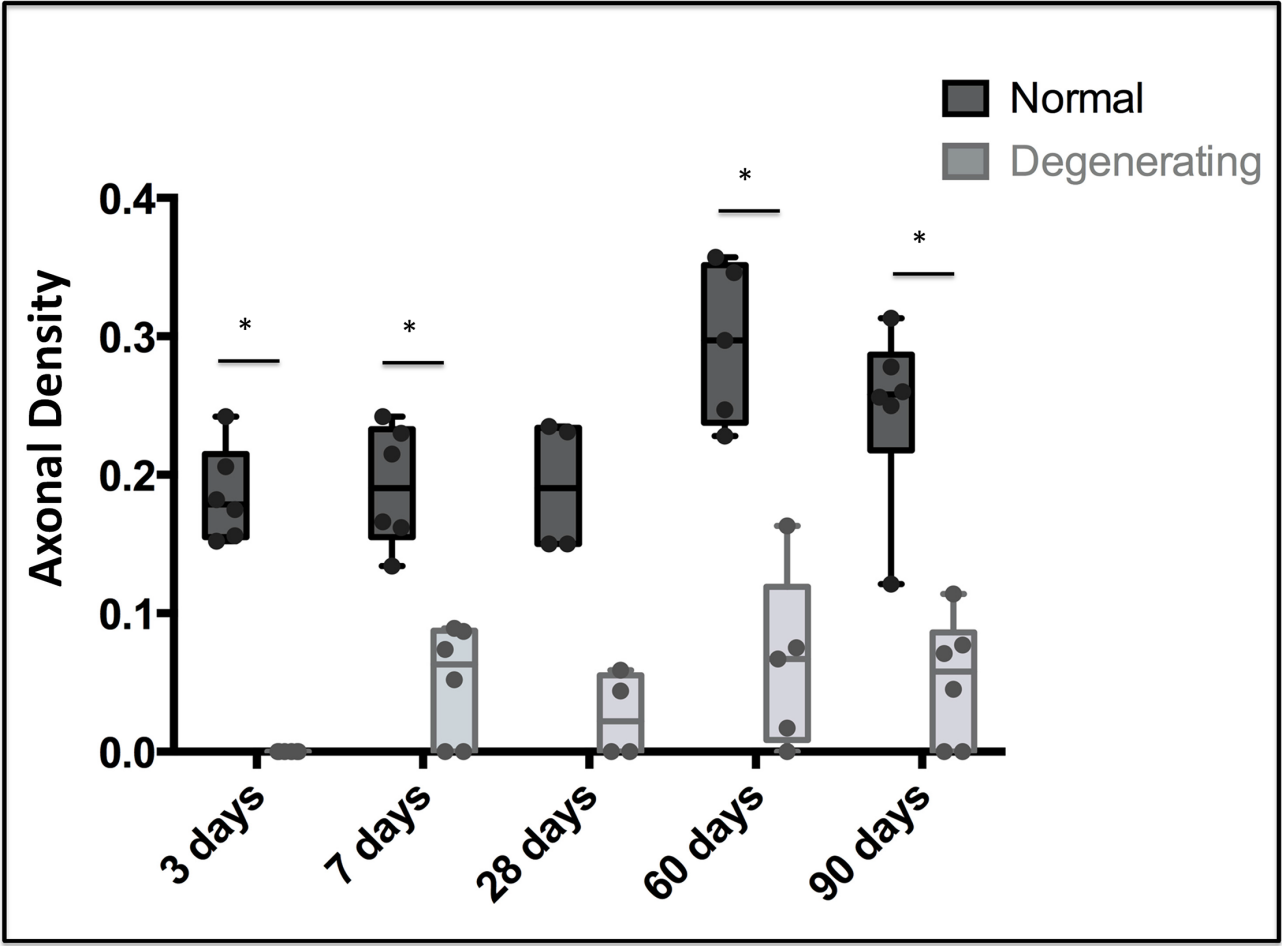
Axonal density of normal and degenerating axons observed in all time points. Boxplot overlaid with sample distribution of axonal density of normal and degenerating axons. * Statistically significant differences (p≤0.05).

Higher values of axonal density were observed in fascicles with normal characteristics compared to degenerating axons at all the time points. Statistical analysis revealed significant differences between normal and degenerating axons at all time points, except 28 dpa. Low numbers of nerves were measured in samples from day 28, which could have affected the precision of the statistical comparisons (Fig 3)(p≤0.05).

**Fig 4:**
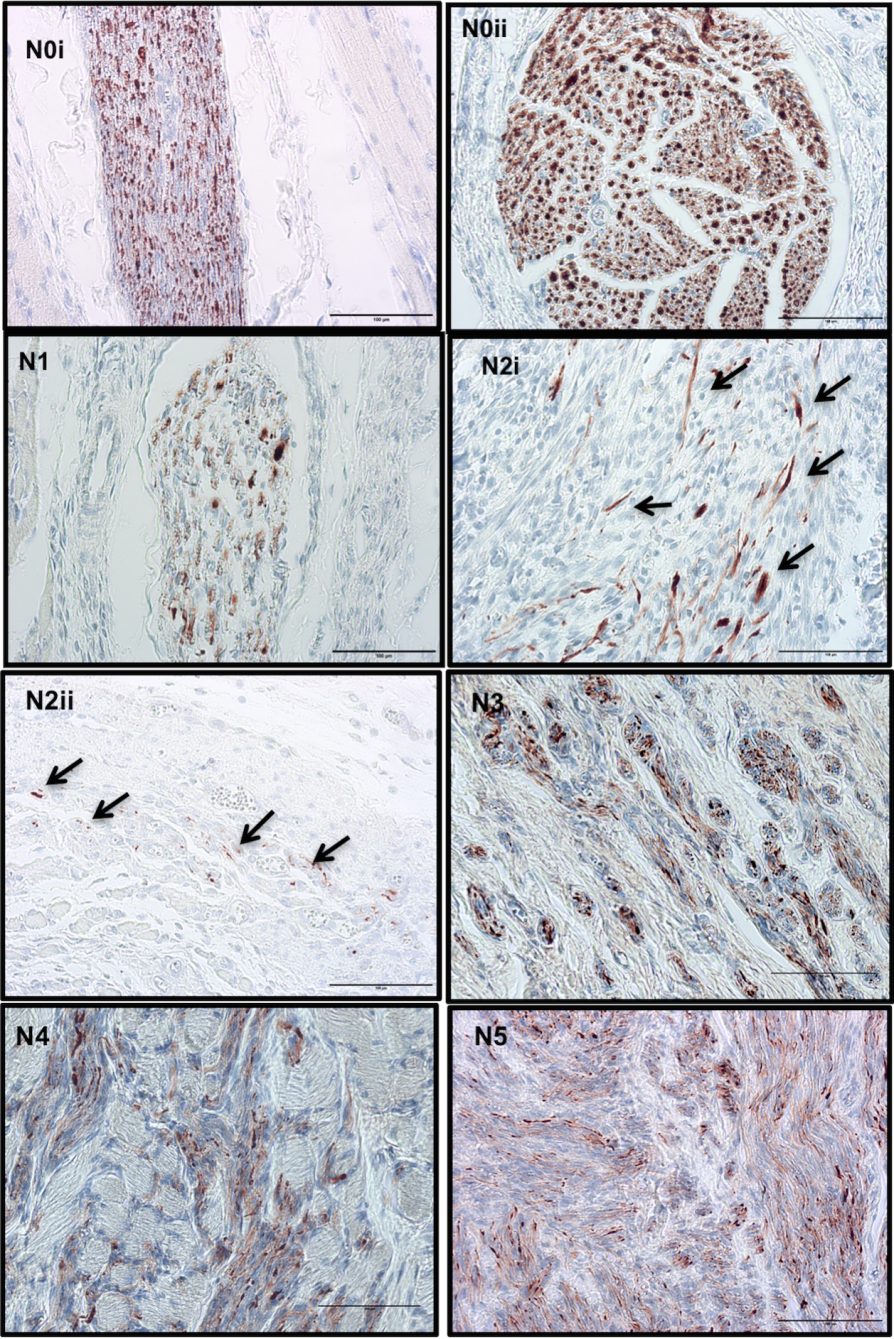
Classification of histological characteristics of nerves in limb stumps after amputation. Normal nerve (N0i: longitudinal and N0ii: transversal section), degenerating axons (N1), axonal sprouts (N2i: at the end of damaged fascicles, arrows show elongated structures corresponding to axonal fibers and N2ii: at distal area, arrows show isolated axons appearing as donut ring-like structures), unorganized axons bundled in connective tissue (N3), unorganized axon ramifications in muscle (N4), neuroma—highly unorganized axons in tissue (N5). Scale bar = 100μm.

Great variability of nerve morphological features was observed with the most relevant listed in the predetermined 6 separate categories (Fig 4). Due to the high variability in the position and orientation of the re-growing nerves in the rat limb stumps, sections contained nerves dissected longitudinally and transversely (Fig 4N0i and 4N0ii).

Normal nerves (N0), when oriented longitudinally, appeared as neatly structured axons running along myelin sheaths, while when sectioned transversally appeared as a ring-like structures formed by compacted axon fibers surrounded by connective tissue forming a neural membrane (Fig 4N0i and 4N0ii). Normal nerves observed in tissue sections appeared to arise from branches of the main nerves of the brachial plexus (ulnar, median and radial), however, in the later time points, these were visible only in the distal area of the stump tissues, closer to the ulnar, median and radial nerve trunks.

Degenerating axons (N1), were identified in nerve fascicles with lower density of NF signal in their structure (Fig 4N1). Axons in the N2 category (axonal sprouts) appeared as delicate and sometimes isolated neurofilaments that looked like donut-rings or fine fibrils within the tissue (Fig 4N2). More precisely, axons showing positive signal should meet the following criteria; not exceed a maximum of 8–10 stripes or dots within an area of 200μm^2^, not be arranged in bundle-like structures and be located a minimum of 100μm distance from a large nerve branch. Unorganized axonal features (N3), consisted of unorganized bundles of axons incorporated in connective tissue, histologically appearing as zones with many axonal fascicles of distinct size and orientation (Fig 4N3); unorganized axons growing into muscle (N4), appeared as small bundles of disorganized axons trapped in muscle (Fig 4N4), usually located in the middle area of the stump tissue, adjacent to the bone, and finally tangled axon fascicles embedded in fibrous tissue (N5) corresponded to a classical neuroma [17,18,32] in late stages of development (Fig 4N5).

**Fig 5:**
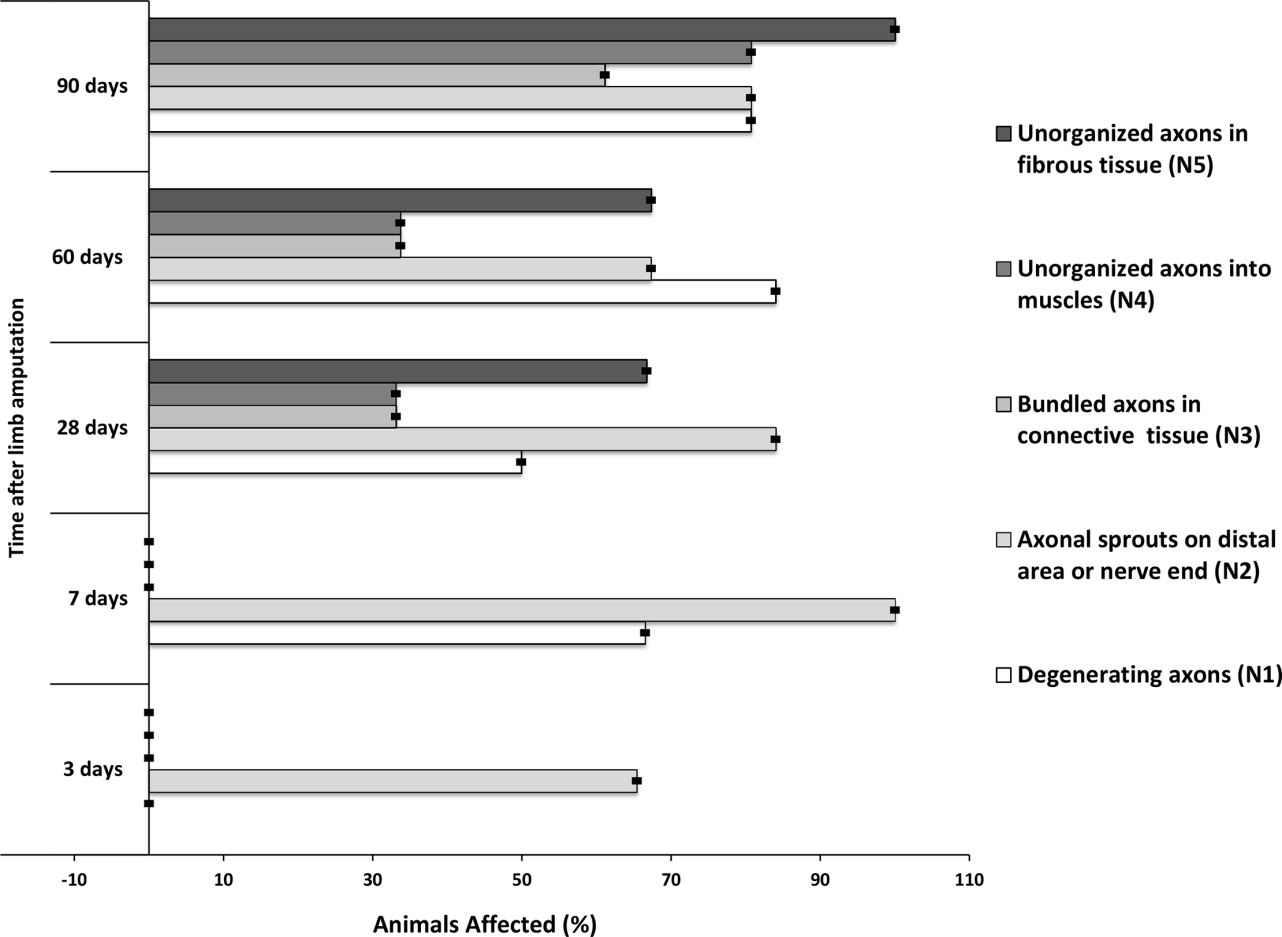
Prevalence of morphological characteristics in rat limb stump tissue analyzed at each time point. Error bars represent confidence interval of 95%.

Present in different areas of the tissue sections, degenerating axons (N1) first appeared at day 7 in approximately 60% of the samples, then their number decreased at 28 days, and again increased at days 60 and 90. Axons in the N2 category were visible in 60% and 100% of samples at day 3, and 7 respectively, and at later time points became less evident (Fig 5). Unorganized features started to appear at 28 days post-amputation, where 33% of the samples presented axons in N3 and N4 stages, while 66% already revealed N5 stage. A similar pattern was observed at 60 days although the incidence of N3, N4, and N5 stages increased at day 90, when 100% of samples showed axonal fascicles in N5 stage. The distribution of morphological characteristics at each time point is shown in Fig 5 and is described in detail below when discussing each time point.

### Nerve regrowth and axonal appearance over time

In samples in early stages after amputation (3 and 7 days) nerve branches from the brachial plexus appeared mainly normal, distributed along the long axis of the limb from proximal to distal areas. In some samples it was even possible to visualize complete fascicles of ulnar, median and/or radial nerves running the entire length of the stump from the humeral head to the distal zones.

In some 3-day sections, where nerve fibers were sectioned, signs of axonal sprouting were already visible in middle and distal areas (Fig 4N2i), and at 7 days, 100% of samples had this appearance. Samples collected at 3 days did not reveal nerve fascicles with lower axonal density (axonal degeneration) in the distal area (closer to injured areas), however the majority of 7-day samples did (Fig 5). Confidence intervals (95% CI) of bootstrapped data varied in very low range indicating a small possible range in the prevalence of the features analyzed.

Twenty-eight days after limb amputation, the 6 nerve characteristics of interest appeared in stump tissues. Nerve fascicles with normal and degeneration characteristics (N0 and N1) showed no significant difference in axonal density values. Apart from these fascicles, axonal sprouts, especially in the distal area, appeared like doughnut-ring structures (N2) (Fig 4N2ii). Also from this time point, zones of unorganized axonal growth were visible along the tissue stump (N3, N4 and N5). Twenty-eight day samples revealed unorganized growth of axonal fibers surrounding normal appearing nerve fascicles. This correlated with zones of nerve enlargement in fascicles sectioned longitudinally. Moreover, in the same amputated limb variability of axonal characteristics and neuroma formation was detected throughout the stump tissue and in zones that anatomically correlates with areas of ulnar, median, radial and musculo-cutaneous nerve fibers.

At days 60 and 90 normal appearing nerves were present in all samples, although gradually less evident and only present in the most proximal areas of the stump, closer to the trunks of nerves originated from the brachial plexus. The incidence of degenerating nerves was higher at days 60 and 90 compared to earlier time points (7 and 28 days). Sixty and 90 day stump tissue contained unorganized bundled axons and fully formed neuromas, which were more evident and prevalent than in 28 day samples. Despite no differences in axonal density among neuromas at the different time points, at 90 days abundant growth of unorganized axons into conjunctive and muscle tissue was visible.

## Discussion

Herein we document the progression of nerve regrowth and neuroma development from the time of injury for 90 days after amputation. Using IHC stain for neurofilament (NF) protein we were able to identify specific characteristics of axons and their relation with surrounding tissues throughout the healing and regenerative process. These neurofilament proteins play important structural roles in neurons; together with microtubules and associated proteins, they sustain the axonal and dendritic branching patterns and promote axonal growth and thickening [33,34]. Variability of nerve patterns observed in our limb stump tissues over time could be correlated with different stages of nerve regrowth and neuroma development. Normal appearing nerve fascicles were visible in all sections analyzed, particularly at the early stages after amputation when their distribution in the stump was near the site of injury. At later time points (60 and 90 days) normal appearing nerve fascicles were only visible at a distance from the injured stump tissue, near the humeral head.

Wallerian degeneration, which is essential for axon regeneration, starts hours after injury with activation of phospholipases expressed in the SCs and macrophages, that promote the removal of myelin and myelin-associated glycoproteins from the injured area (reviewed in [35,36]). After debris clearing, growth factors are produced to stimulate Schwann cell migration and axonal regrowth (reviewed in [37]).

In this study, the presence of fascicles with less evident NF signal directly correlated with low axonal density, which was indicative of degenerating nerves in the stump tissues. Using epifluorescence microscopy, alterations at the axonal ultra-structure level have been reported in rat optical nerves already 30 minutes after injury [38]. Although the techniques used in the present study were not sensitive enough to detect alterations at this level, we could detect degenerating axons from day 7 on. Other models and/or microscopy techniques would have been necessary to identify these minimal changes in nerve structures.

Intrinsic mechanisms involving calcium influx and activation of the protease Calpain are responsible for cytoskeletal degradation and axon degeneration (reviewed in [39]). In addition, NF protein becomes sensitive to proteases when they are dephosphorylated (reviewed in [34]). Nerve injury significantly increases the levels of dephosphorylated Nuclear Factor of Activated T-cells—NFATc4 [40]. Protection mechanisms against phosphatases have also been shown to play an important role in nerve regeneration [41,42]. The process of axonal degeneration has been observed not only in traumatic injuries, but also as a consequence of degenerative diseases. Despite the fact that the mechanism of this process is not fully understood, studies suggest similarities between both phenomena (reviewed in [43]). Delayed axonal degeneration could also be associated with the expression of “Wallerian degeneration slow” (WldS) protein [44], impairment of Nmnat2 proteins in the cell body [45], and the absence of trophic signals to neurons [46]. In our study, degenerating axons seen in 90 dpa samples, might not be directly related to Wallerian degeneration, that occurs immediately after injury, however may be associated with other environmental factors and/or these suggested mechanisms instead. As a consequence of the lack of NF, deficiency in nerve regeneration can occur, although this may not be the result of neuronal death following injury [47].

Signs of axonal sprouts were visible at the site of amputation where the nerve was cut already at day 3 and more so at day 7 post amputation. Spinal cord injuries initially present minimal axon degeneration followed by nerve regeneration from the proximal axons towards the distal organ [48]. The axonal sprouts we saw early on could be attempts by axons to begin regenerating after Wallerian degeneration. Dun & Parkinson described transected mouse sciatic nerve axons that showed rapid axonal regrowth from day 4, leading to “misdirected axonal growth of 1.46 mm” on day 7 [49]. Studies have shown that after injury, local growth factor and protein production support axonal growth and promotes nerve repair [8,50]. Although we did not measure protein levels, increased local protein production and the beginning of axonal attempts to regenerate could have induced the sprout growth we observed in our IHC sections. Our control samples revealed normal nerve fascicles with no sprouts, suggesting that the sprouts observed at days 3 and 7 were axonal regrowth and not artifacts from the cutting technique.

In this study we observed the first signs of neuroma formation at 28 days post amputation. Two modes of axonal growth in the early stages are suggested—elongation, in which axons advance fast and straight, and—branching, in which numerous small lateral outgrowths are formed [48]. Both elongated and branching structures were visible in our stump tissue at days 3 and 7. At 28 days we observed abnormal bulbous shaped axonal structures growing into muscle and/or fibrotic tissue. In the absence of a guiding structure, the signals that induce directed axon growth persist and instead of re-growing in an organized directional pattern regenerating axons may regrow in unorganized patterns and form neuroma (reviewed in [51]). In our samples, neuroma formation was detected already at 28 days, and became more evident and widespread at later time points (60 and 90 days).

In 28- and 60-day stumps areas of unorganized axonal structures were observed side-by-side with normal fascicles. Literature describes cases of segmented nerve injury whereby axons from the proximal end of a severed nerve regrow towards the distal end through tunnel-like structures formed by Schwann cells [48]. Since in our amputation limb model the distal nerve segment is missing, Schwann cells are not able to form tunnels to guide the axons, resulting in unorganized nerve growth. Thus the neuromas we observed in 28 dpa stump tissue could have been caused by the absence of these Schwann cell tunnels and signaling, whereby attempts to communicate with the target organ to establish axonal recovery did not occur.

In parallel with nerve signaling and attempts to regrow, fibrotic tissue is formed in order to repair and close the wound. It is hypothesized that during axonal regrowth regenerating nerve fibers and associated connective tissue extend into regions at the same time that severely damaged tissue is in a proliferative phase of healing by secondary intention. When blocked by fibrotic tissue, the regenerating neural axons form spirals and end discs and become irregularly dispersed throughout the connective tissue [15]. The histologic changes we observed in our stump tissues over time are consistent with the above-described hypothesis for neuroma formation proposed by Foltán et al. (2008). Growth of regenerative nerve fibers into connective tissue is represented in our N3 stage samples, while nerve regrowth into damaged tissue, muscle in our case, corresponds to our N4 stage samples. Finally, axons blocked by fibrotic tissues forming massive chaotic structures, i.e. neuroma, corresponds to the N5 stage of our scale.

Traumatic neuroma are frequently characterized by a massive tumor showing irregular arrangement of nerve fascicles within a collagenous and fibroblastic stroma [19,52–55]. Moreover, these lesions frequently present incomplete encapsulation and a mix of axons and Schwann cells [17]. These diagnostic descriptions of neuroma coincide with our N5 stage (unorganized axonal growth into fibrotic tissue) classification of fully formed neuroma.

Detailed histological analysis of symptomatic and asymptomatic neuromas have shown that fascicle number and diameter and nerve tissue density cannot be used to distinguish between painful and non-painful neuromas [53]. Histological signs of chronic inflammation have been shown to be associated with painful neuromas [18]. However, pain and abnormal sensitivity can also occur in the absence of inflammation. Although our study did not focus on “painful” neuromas, we frequently observed unorganized nerve regrowth into connective tissue and muscle. Weng et al, identified the expression of alpha smooth muscle actin (α-SMA) in painful neuromas [52,56]. Alfa-SMA is a phenotypic marker for myofibroblast activity, which contribute to increased contractile activity of myofibroblasts [57]. Based on this it is conceivable that nerve fibers that attempt to regrow into/through muscle could originate painful neuroma.

The histological characteristics we observed in this study; unorganized axon bundles, axonal ingrowth into muscles and fibrous tissue, could be the manifestation of different stages of neuroma formation. We observed that axon regrowth starts already 3 days after amputation and by 28 days neuroma formation is clearly visible in many different nerves present in the stump tissue. Signals for axonal regrowth appear to be activated at 60 and 90 days. Moreover in this study, where there was no intervention to prevent neuroma formation, by day 90 post-amputation neuroma formation occurred in 100% of the cases evaluated.

Our findings indicate that 28 days is a critical time point at which neuroma formation has already occurred. Based on this we propose that treatments focused on inhibiting neuroma development should be initiated prior to this critical time point, when growth is still dynamic and can be influenced. Future research will focus on studying the underlying mechanisms at work during this early time period in order to target specific factors that influence neuroma formation in the dynamic early stages of development. This could lead to new and better treatments and/or prevention strategies to alleviate suffering in these patients.

## Supporting information

S1 Fig Representative images showing features of general morphology of ulnar, median and radial nerves branches stained with AB&OG. (A, B, C and D) Longitudinal and transversal cut of nerve brunches demonstrating normal features (N0), degenerating axons (N1) and/or axonal sprouts (N2). Nerves with advanced pathological features: (E) unorganized axons bundled in connective tissue (N3), (F) unorganized axon ramifications in muscle (N4) and (G) neuroma—highly unorganized axons in tissue (N5). All images have 20x magnification with scale bar = 100μm.
(PDF)

## Abbreviations

α-SMA: Alfa Smooth Muscle ActinAB&OG, Alcian Blue/Orange G-Hematoxilin-Eosin
BDNF: Brain Derived Nerve Factor
DPA: Days Post Amputation
IHC: Immunohistochemical
NF: Neurofilament protein
NGF: Nerve Growth Factor
NMDA: N-methyl-D-aspartate
ROI: Region of Interest

## References

[1] Horgan O, MacLachlan M. Psychosocial adjustment to lower-limb amputation: A review. *Disability and rehabilitation* 2004; 26 (14–15): 837–850. 15497913 10.1080/09638280410001708869

[2] Hisam A, Ashraf F, Rana MN, Waqar Y, Karim S, Irfan F. Health Related Quality of Life in Patients with Single Lower Limb Amputation. *Journal of the College of Physicians and Surgeons—Pakistan*: *JCPSP* 2016; 26 (10): 851–854. 27806816

[3] Buchheit T, Van de Ven Thomas, Hsia H-LJ, McDuffie M, MacLeod DB, White W, et al. Pain Phenotypes and Associated Clinical Risk Factors Following Traumatic Amputation: Results from Veterans Integrated Pain Evaluation Research (VIPER). *Pain medicine (Malden*, *Mass*.*)* 2016; 17 (1): 149–161; 10.1111/pme.12848 26177330 PMC6280998

[4] Rajput K, Reddy S, Shankar H. Painful neuromas. *The Clinical journal of pain* 2012; 28 (7): 639–645; 10.1097/AJP.0b013e31823d30a2 22699131

[5] van der Avoort DJJC, Hovius SER, Selles RW, van Neck JW, Coert JH. The incidence of symptomatic neuroma in amputation and neurorrhaphy patients. *Journal of plastic*, *reconstructive & aesthetic surgery*: *JPRAS* 2013; 66 (10): 1330–1334; 10.1016/j.bjps.2013.06.019 23845907

[6] Kryger GS, Kryger Z, Zhang F, Shelton DL, Lineaweaver WC, Buncke HJ. Nerve growth factor inhibition prevents traumatic neuroma formation in the rat. *The Journal of hand surgery* 2001; 26 (4): 635–644; 10.1053/jhsu.2001.26035 11466637

[7] Marcol W, Kotulska K, Larysz-Brysz M, Kowalik JL. BDNF contributes to animal model neuropathic pain after peripheral nerve transection. *Neurosurgical review* 2007; 30 (3): 235–43; discussion 243; 10.1007/s10143-007-0085-5 17530308

[8] Gillen C, Korfhage C, Müller HW. ■ REVIEW: Gene Expression in Nerve Regeneration. *Neuroscientist* 2016; 3 (2): 112–122; 10.1177/107385849700300210

[9] Yamauchi J, Chan JR, Shooter EM. Neurotrophins regulate Schwann cell migration by activating divergent signaling pathways dependent on Rho GTPases. *Proceedings of the National Academy of Sciences of the United States of America* 2004; 101 (23): 8774–8779; 10.1073/pnas.0402795101 15161978 PMC423271

[10] Michalski B, Bain JR, Fahnestock M. Long-term changes in neurotrophic factor expression in distal nerve stump following denervation and reinnervation with motor or sensory nerve. *Journal of neurochemistry* 2008; 105 (4): 1244–1252; 10.1111/j.1471-4159.2008.05224.x 18194437 PMC3414532

[11] Wood MD, Mackinnon SE. Pathways regulating modality-specific axonal regeneration in peripheral nerve. *Experimental neurology* 2015; 265: 171–175; 10.1016/j.expneurol.2015.02.001 25681572 PMC4399493

[12] Namgung U. The role of Schwann cell-axon interaction in peripheral nerve regeneration. *Cells*, *tissues*, *organs* 2014; 200 (1): 6–12; 10.1159/000370324 25765065

[13] Chan KM, Gordon T, Zochodne DW, Power HA. Improving peripheral nerve regeneration: From molecular mechanisms to potential therapeutic targets. *Experimental neurology* 2014; 261: 826–835; 10.1016/j.expneurol.2014.09.006 25220611

[14] Faroni A, Mobasseri SA, Kingham PJ, Reid AJ. Peripheral nerve regeneration: Experimental strategies and future perspectives. *Advanced drug delivery reviews* 2015; 82–83: 160–167; 10.1016/j.addr.2014.11.010 25446133

[15] Foltán R, Klíma K, Spacková J, Sedý J. Mechanism of traumatic neuroma development. *Medical hypotheses* 2008; 71 (4): 572–576; 10.1016/j.mehy.2008.05.010 18599222

[16] Rainsbury JW, Whiteside OJH, Bottrill ID. Traumatic facial nerve neuroma following mastoid surgery: A case report and literature review. *The Journal of laryngology and otology* 2007; 121 (6): 601–605; 10.1017/S0022215106004993 17140460

[17] Argenyi ZB. Immunohistochemical characterization of palisaded, encapsulated neuroma. *Journal of cutaneous pathology* 1990; 17 (6): 329–335. 1705947 10.1111/j.1600-0560.1990.tb00108.x

[18] Vora AR, Bodell SM, Loescher AR, Smith KG, Robinson PP, Boissonade FM. Inflammatory cell accumulation in traumatic neuromas of the human lingual nerve. *Archives of oral biology* 2007; 52 (1): 74–82; 10.1016/j.archoralbio.2006.08.015 17097599

[19] Hirose T, Tani T, Shimada T, Ishizawa K, Shimada S, Sano T. Immunohistochemical demonstration of EMA/Glut1-positive perineurial cells and CD34-positive fibroblastic cells in peripheral nerve sheath tumors. *Modern pathology*: *an official journal of the United States and Canadian Academy of Pathology*, *Inc* 2003; 16 (4): 293–298; 10.1097/01.MP.0000062654.83617.B7 12692193

[20] Egami S, Tanese K, Honda H, Kasai H, Yokoyama T, Sugiura M.Traumatic neuroma on the digital tip: Immunohistochemical analysis of inflammatory signaling pathways. *The Journal of dermatology* 2016; 43 (7): 836–837; 10.1111/1346-8138.13297 26876467

[21] Khan J, Noboru N, Young A, Thomas D. Pro and anti-inflammatory cytokine levels (TNF-α, IL-1β, IL-6 and IL-10) in rat model of neuroma. *Pathophysiology*: *the official journal of the International Society for Pathophysiology* 2017; 24 (3): 155–159; 10.1016/j.pathophys.2017.04.001 28462800

[22] Yan H, Zhang F, Kolkin J, Wang C, Xia Z, Fan C. Mechanisms of nerve capping technique in prevention of painful neuroma formation. *PloS one* 2014; 9 (4): e93973; 10.1371/journal.pone.0093973 24705579 PMC3976365

[23] Hsu E, Cohen SP. Postamputation pain: Epidemiology, mechanisms, and treatment. *Journal of pain research* 2013; 6: 121–136; 10.2147/JPR.S32299 23426608 PMC3576040

[24] Stokvis A, Coert JH, van Neck JW. Insufficient pain relief after surgical neuroma treatment: Prognostic factors and central sensitisation. *Journal of plastic*, *reconstructive & aesthetic surgery*: *JPRAS* 2010; 63 (9): 1538–1543; 10.1016/j.bjps.2009.05.036 19559663

[25] Pet MA, Ko JH, Friedly JL, Mourad PD, Smith DG. Does targeted nerve implantation reduce neuroma pain in amputees? *Clinical orthopaedics and related research* 2014; 472 (10): 2991–3001; 10.1007/s11999-014-3602-1 24723142 PMC4160473

[26] Thomsen L, Schlur C. Incidence des douleurs névromateuses après manchonnage par un tube de collagène des sutures nerveuses directes. Étude prospective de 185 cas [Incidence of painful neuroma after end-to-end nerve suture wrapped into a collagen conduit. A prospective study of 185 cases]. *Chirurgie de la main* 2013; 32 (5): 335–340; 10.1016/j.main.2013.07.001 24075502

[27] Ay S, Akinci M. Primary transposition of digital nerves into muscle in second ray amputation: A possible answer for neuroma formation. *Techniques in hand & upper extremity surgery* 2003; 7 (3): 114–118. 16518229 10.1097/00130911-200309000-00008

[28] Ismail Hossam El-Din Ali, Kasem MA, Mostafa FE-H. Influence of Blood Flow on the Neuroma Formation after Transposition of the Nerve Stump into Vein: Experimental and Clinical Study. *Journal of hand and microsurgery* 2017; 9 (1): 17–27; 10.1055/s-0037-1602126 28442857 PMC5403727

[29] Economides JM, DeFazio MV, Attinger CE, Barbour JR. Prevention of Painful Neuroma and Phantom Limb Pain After Transfemoral Amputations Through Concomitant Nerve Coaptation and Collagen Nerve Wrapping. *Neurosurgery* 2016; 79 (3): 508–513; 10.1227/NEU.0000000000001313 27306717

[30] Yao C, Zhou X, Zhao B, Sun C, Poonit K, Yan H.Treatments of traumatic neuropathic pain: A systematic review. *Oncotarget* 2017; 8 (34): 57670–57679; 10.18632/oncotarget.16917 28915703 PMC5593675

[31] Nowalk JR, Flick LM. Visualization of Different Tissues Involved in Endochondral Ossification With Alcian Blue Hematoxylin and Orange G/Eosin Counterstain. *journal of histotechnology* 2008; 31 (1): 19–21; 10.1179/014788808794748494

[32] Leppik LP, Froemel D, Slavici A, Ovadia ZN, Hudak L, Henrich D, et al Effects of electrical stimulation on rat limb regeneration, a new look at an old model. *Scientific reports* 2015; 5: 18353; 10.1038/srep18353 26678416 PMC4683620

[33] Helfand BT, Mendez MG, Pugh J, Delsert C, Goldman RD. A role for intermediate filaments in determining and maintaining the shape of nerve cells. *Molecular biology of the cell* 2003; 14 (12): 5069–5081; 10.1091/mbc.E03-06-0376 14595112 PMC284808

[34] Yuan A, Rao MV, Veeranna, Nixon RA. Neurofilaments and Neurofilament Proteins in Health and Disease. *Cold Spring Harbor perspectives in biology* 2017; 9 (4); 10.1101/cshperspect.a018309 28373358 PMC5378049

[35] Faroni A, Mobasseri SA, Kingham PJ, Reid AJ. Peripheral nerve regeneration: experimental strategies and future perspectives. *Advanced drug delivery reviews* 2015; 82–83: 160–167; 10.1016/j.addr.2014.11.010 25446133

[36] Glenn TD, Talbot WS. Signals regulating myelination in peripheral nerves and the Schwann cell response to injury. *Current opinion in neurobiology* 2013; 23 (6): 1041–1048; 10.1016/j.conb.2013.06.010 23896313 PMC3830599

[37] Gaudet AD, Popovich PG, Ramer MS. Wallerian degeneration: Gaining perspective on inflammatory events after peripheral nerve injury. *Journal of neuroinflammation* 2011; 8: 110; 10.1186/1742-2094-8-110 21878126 PMC3180276

[38] Knöferle J, Koch JC, Ostendorf T, Michel U, Planchamp V, Vutova P, et al Mechanisms of acute axonal degeneration in the optic nerve in vivo. *Proceedings of the National Academy of Sciences of the United States of America* 2010; 107 (13): 6064–6069; 10.1073/pnas.0909794107 20231460 PMC2851885

[39] Wang JT, Medress ZA, Barres BA. Axon degeneration: molecular mechanisms of a self-destruction pathway. *The Journal of cell biology* 2012; 196 (1): 7–18; 10.1083/jcb.201108111 22232700 PMC3255986

[40] Cai Y-Q, Chen S-R, Pan H-L. Upregulation of nuclear factor of activated T-cells by nerve injury contributes to development of neuropathic pain. *The Journal of pharmacology and experimental therapeutics* 2013; 345 (1): 161–168; 10.1124/jpet.112.202192 23386250 PMC3608445

[41] Abe N, Cavalli V. Nerve injury signaling. *Current opinion in neurobiology* 2008; 18 (3): 276–283; 10.1016/j.conb.2008.06.005 18655834 PMC2633416

[42] Mesfin MN, Reyn CR v., Mott RE, Putt ME, Meaney DF. In vitro stretch injury induces time- and severity-dependent alterations of STEP phosphorylation and proteolysis in neurons. *Journal of neurotrauma* 2012; 29 (10): 1982–1998; 10.1089/neu.2011.2253 22435660 PMC3390986

[43] Wang JT, Medress ZA, Barres BA. Axon degeneration: Molecular mechanisms of a self-destruction pathway. *The Journal of cell biology* 2012; 196 (1): 7–18; 10.1083/jcb.201108111 22232700 PMC3255986

[44] Hoopfer ED, McLaughlin T, Watts RJ, Schuldiner O, O'Leary DDM, Luo L. Wlds protection distinguishes axon degeneration following injury from naturally occurring developmental pruning. *Neuron* 2006; 50 (6): 883–895; 10.1016/j.neuron.2006.05.013 16772170

[45] Gilley J, Coleman MP. Endogenous Nmnat2 is an essential survival factor for maintenance of healthy axons. *PLoS biology* 2010; 8 (1): e1000300; 10.1371/journal.pbio.1000300 20126265 PMC2811159

[46] Nikolaev A, McLaughlin T, O'Leary DDM, Tessier-Lavigne M. APP binds DR6 to trigger axon pruning and neuron death via distinct caspases. *Nature* 2009; 457 (7232): 981–989; 10.1038/nature07767 19225519 PMC2677572

[47] Zhu Q, Couillard-Després S, Julien JP. Delayed maturation of regenerating myelinated axons in mice lacking neurofilaments. *Experimental neurology* 1997; 148 (1): 299–316; 10.1006/exnr.1997.6654 9398473

[48] Kerschensteiner M, Schwab ME, Lichtman JW, Misgeld T. In vivo imaging of axonal degeneration and regeneration in the injured spinal cord. *Nature medicine* 2005; 11 (5): 572–577; 10.1038/nm1229 15821747

[49] Dun X-p, Parkinson DB. Visualizing peripheral nerve regeneration by whole mount staining. *PloS one* 2015; 10 (3): e0119168; 10.1371/journal.pone.0119168 25738874 PMC4349735

[50] Verma P, Chierzi S, Codd AM, Campbell DS, Meyer RL, Holt CE, et al Axonal protein synthesis and degradation are necessary for efficient growth cone regeneration. *The Journal of neuroscience*: *the official journal of the Society for Neuroscience* 2005; 25 (2): 331–342; 10.1523/JNEUROSCI.3073-04.2005 15647476 PMC3687202

[51] Wu Di, Murashov AK. Molecular mechanisms of peripheral nerve regeneration: Emerging roles of microRNAs. *Frontiers in physiology* 2013; 4: 55; 10.3389/fphys.2013.00055 23554595 PMC3612692

[52] Yan H, Gao W, Pan Z, Zhang F, Fan C. The expression of α-SMA in the painful traumatic neuroma: Potential role in the pathobiology of neuropathic pain. *Journal of neurotrauma* 2012; 29 (18): 2791–2797; 10.1089/neu.2012.2502 23020218

[53] Vora AR, Loescher AR, Craig GT, Boissonade FM, Robinson PP. A light microscopical study on the structure of traumatic neuromas of the human lingual nerve. *Oral surgery*, *oral medicine*, *oral pathology*, *oral radiology*, *and endodontics* 2005; 99 (4): 395–403; 10.1016/j.tripleo.2004.08.011 15772589

[54] Mavrogenis AF, Pavlakis K, Stamatoukou A, Papagelopoulos PJ, Theoharis S, Zoubos AB, et al Current treatment concepts for neuromas-in-continuity. *Injury* 2008; 39 Suppl 3: S43–8; 10.1016/j.injury.2008.05.015 18715561

[55] Kang J, Yang P, Zang Q, He X. Traumatic neuroma of the superficial peroneal nerve in a patient: A case report and review of the literature. *World journal of surgical oncology* 2016; 14 (1): 242; 10.1186/s12957-016-0990-6 27613606 PMC5018173

[56] Weng W, Zhao B, Lin D, Gao W, Li Z, Yan H. Significance of alpha smooth muscle actin expression in traumatic painful neuromas: A pilot study in rats. *Scientific reports* 2016; 6: 23828; 10.1038/srep23828 27021914 PMC4810523

[57] Hinz B, Celetta G, Tomasek JJ, Gabbiani G, Chaponnier C. Alpha-smooth muscle actin expression upregulates fibroblast contractile activity. *Molecular biology of the cell* 2001; 12 (9): 2730–2741. 10.1091/mbc.12.9.2730 11553712 PMC59708

