# Supplementary material for: Time course of traumatic neuroma development
Source: PLoS One. 2018 Jul 16;13(7):e0200548. doi: 10.1371/journal.pone.0200548 (PMC6047790; doi:10.1371/journal.pone.0200548)
Supplement: S1 Fig — (A, B, C and D) Longitudinal and transversal cut of nerve brunches demonstrating normal features (N0), degenerating axons (N1) and/or axonal sprouts (N2). Nerves with advanced pathological features: (E) unorganized axons bundled in connective tissue (N3), (F) unorganized axon ramifications in muscle (N4) and (G) neuroma—highly unorganized axons in tissue (N5). All images have 20x magnification with scale bar = 100μm. (PDF) [file pone.0200548.s001.pdf]

S1 Supporting Information

S1 Figure

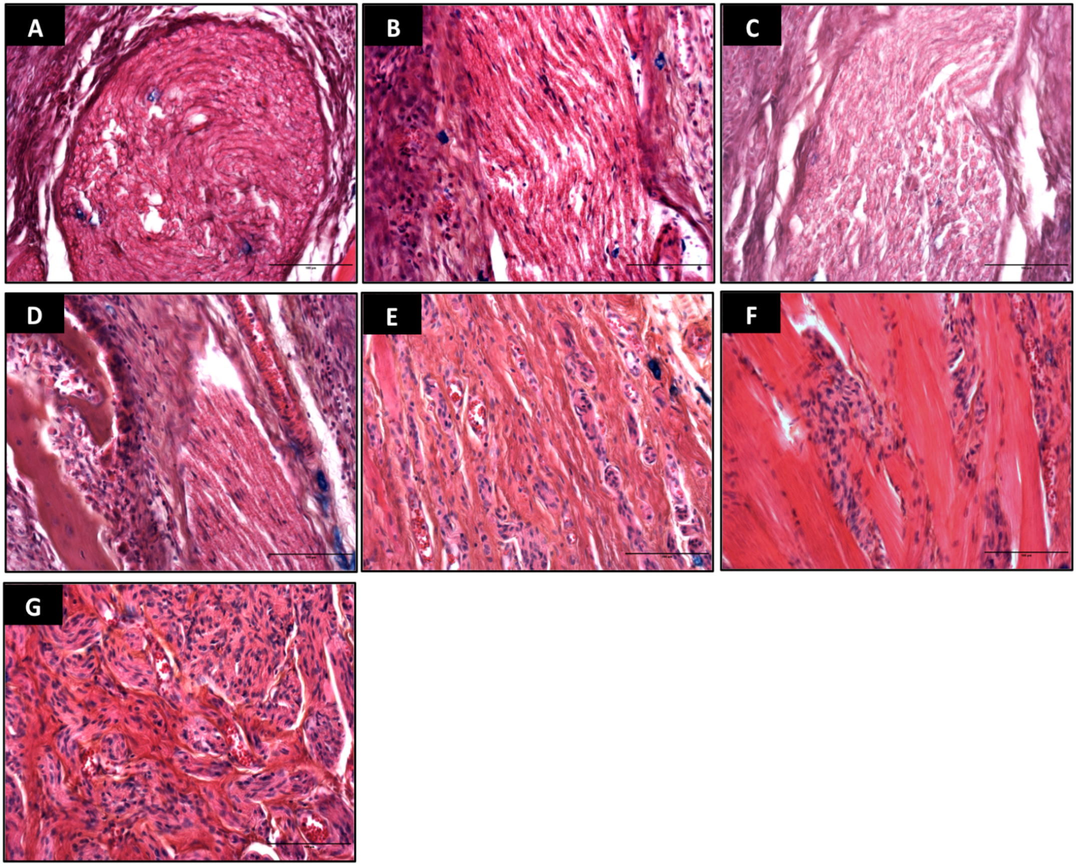

Fig S1 – **Representative images showing features of general morphology of ulnar, median and radial nerves branches stained with AB&OG**. (A, B, C and D) Longitudinal and transversal cut of nerve brunches demonstrating normal features (N0), degenerating axons (N1) and/or axonal sprouts (N2). Nerves with advanced pathological features: (E) unorganized axons bundled in connective tissue (N3), (F) unorganized axon ramifications in muscle (N4) and (G) neuroma - highly unorganized axons in tissue (N5). All images have 20x magnification with scale bar = 100μm.
